# Design and Imaging of Ground-Based Multiple-Input Multiple-Output Synthetic Aperture Radar (MIMO SAR) with Non-Collinear Arrays

**DOI:** 10.3390/s17030598

**Published:** 2017-03-15

**Authors:** Cheng Hu, Jingyang Wang, Weiming Tian, Tao Zeng, Rui Wang

**Affiliations:** 1 School of Information and Electronics, Beijing Institute of Technology, Beijing 100081, China; hucheng.bit@gmail.com (C.H.); ryan1312@foxmail.com (J.W.); zengtao@bit.edu.cn (T.Z.); bit.wangrui@gmail.com (R.W.); 2 Beijing Key Laboratory of Embedded Real-Time Information Processing Technology, Beijing 100081, China

**Keywords:** MIMO radar, MIMO imaging, near-field imaging, height difference between T/R arrays, grating lobes

## Abstract

Multiple-Input Multiple-Output (MIMO) radar provides much more flexibility than the traditional radar thanks to its ability to realize far more observation channels than the actual number of transmit and receive (T/R) elements. In designing the MIMO imaging radar arrays, the commonly used virtual array theory generally assumes that all elements are on the same line. However, due to the physical size of the antennas and coupling effect between T/R elements, a certain height difference between T/R arrays is essential, which will result in the defocusing of edge points of the scene. On the other hand, the virtual array theory implies far-field approximation. Therefore, with a MIMO array designed by this theory, there will exist inevitable high grating lobes in the imaging results of near-field edge points of the scene. To tackle these problems, this paper derives the relationship between target’s point spread function (PSF) and pattern of T/R arrays, by which the design criterion is presented for near-field imaging MIMO arrays. Firstly, the proper height between T/R arrays is designed to focus the near-field edge points well. Secondly, the far-field array is modified to suppress the grating lobes in the near-field area. Finally, the validity of the proposed methods is verified by two simulations and an experiment.

## 1. Introduction

Ground-based Synthetic Aperture Radar (GB-SAR) is a kind of radar system which can realize two-dimensional high-resolution imaging by linear motion on a slide rail with synthetic aperture technology [1,2,3,4]. It is widely used in the field of slope monitoring [5,6] due to its ability to measure tiny deformations accurately with differential interferometry technology. Nevertheless, because of its need for a slide, the radar system, which is difficult to move with its complex structure, has very high terrain flatness requirements. Meanwhile, the system’s imaging interval is too long to measure vibrations.

In recent years, multiple-input multiple-output (MIMO) technology featuring multi-antenna structures has been introduced into the radar field [7,8,9]. By applying the waveform diversity technique, it can obtain far more observation channels and degrees of freedom than the actual number of transmit and receive (T/R) elements. MIMO radars can be divided into two modes by the location of T/R elements relative to the observed target: collocated and statistical [10]. Essentially, radar imaging is the focus of coherent data on a certain observation aperture, thus MIMO imaging radars generally adopt the collocated mode [11], whose T/R elements concentrate on the same observation angle of the target.

Without any spatial resolution loss, MIMO imaging radar outperforms the traditional GB-SAR with its high temporal resolution and freedom from motion compensation problems. Moreover, this key advantage provides a basis for vibration measurements. Therefore, researchers in many different fields have been attracted by MIMO radar imaging technology [12,13,14,15,16,17], such as security, medical imaging, slope monitoring [17,18,19], vehicle orientation estimation [20] and through-wall imaging [21].

To achieve good imaging performance, MIMO arrays are usually designed by the virtual array theory [22], which equates the actual array to a one-dimensional uniform linear array (ULA). Obviously, that all T/R elements should be on the same line is an implied requirement of this theory. Considering the physical size of actual antennas and the coupling effect between T/R elements, this assumption cannot be realized in practical systems. In fact, a certain height difference between T/R arrays of a MIMO radar is unavoidable. In other words, the MIMO radar array is a non-collinear array. This height difference does not affect the imaging performance of far-field targets after calibration and thus can be ignored, which has been verified in [17] by an experiment using an array with 0.3 m height difference. For near-field targets, the effect is not obvious either if the azimuth angle and elevation angle of the scene are small enough. For example, the arrays in [22,23] also have a height difference, but neither the 2D imaging results nor the 3D imaging results with synthetic aperture technology were affected by it due to the small scenes. However, the height difference will defocus edge points (points with maximum azimuth angle and elevation angle) in the near-field area and cannot be ignored in slope monitoring radar whose scene is with a large angle and wide depth. Unfortunately, nowadays there is no research addressing this problem. 

In addition, high grating lobes will appear in the imaging results obtained in the near-field area, even with collinear T/R arrays, as the virtual array is no longer uniform in this case. Inspired by the relationship between the position of grating lobes and the frequency, Zhuge used ultra-wideband (UWB) technology to smooth the grating lobes [13,24,25], showing that grating lobes won’t appear when the relative bandwidth is 150% with the center frequency of 11 GHz. Gumbmann proposed a method to suppress grating lobes by changing the positions of transmit elements (TEs) [12,23]. This method is shown to be valid to a certain extent by simulations and experiments, but cannot support to design an array with a certain grating lobe requirement.

This paper discusses the above two problems in near-field imaging and considers two aspects. On the one hand, the bad imaging performance of the near-field edge points is explained by the peak position’s offset of transmit pattern. Furthermore, a design method based on height difference is proposed to satisfy the specifications. On the other hand, the appearance of grating lobes is explained by the distortion of T/R patterns. With this explanation, a design criterion of MIMO arrays with low grating lobes is presented.

The structure of this paper is as follows: Section 2 introduces the basic theory of array imaging radar, including virtual array theory and far-field condition for array antennas. Section 3 discusses field division of MIMO arrays and the definition of near-field patterns. The first subsection of Section 4 explains the defocus of edge points in the near-field area and proposes a method to design the height difference between T/R arrays using the peak side lobe rate (PSLR) specifications. Then the second subsection of Section 4 explains the appearance of high grating lobes of edge points and illustrates a method to design MIMO arrays with low grating lobes. Section 5 proves the proposed methods by two simulations and an experiment. Section 6 draws the conclusions.

## 2. Basic Theory of Array Imaging Radar

### 2.1. Virtual Array Theory of MIMO Radar

**Figure 1:**
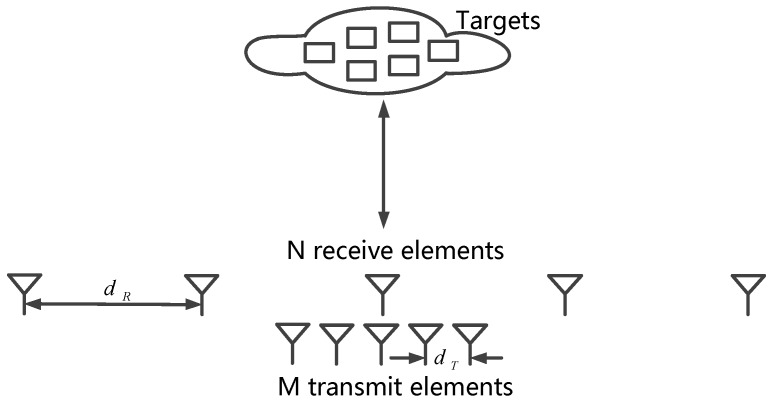
MIMO imaging radar array.

As shown in Figure 1, the MIMO imaging radar with *M* TEs and *N* REs can provide *MN* individual observation echoes from the targets. The virtual array theory demonstrates that every MIMO array can be considered equivalent to a virtual array whose one-way beam pattern is identical to a two-way pattern of the initial array [25]. It can be treated as a receive aperture with the object illuminated by a single transmitter. Denoting the position vectors of the m-th TE (transmit element) and the n-th RE (receive element) as $r_{\mathrm{Tm}}$ and $r_{\mathrm{Rn}}$, respectively, the phase delay of each echo is the interacted result of the positions of TE and RE, which equals to the phase of the signal from a virtual element with the position vector $r_{\mathrm{Tm}}+r_{\mathrm{Rn}}$. Therefore, the processed signals of MIMO array are the same with those of an equivalent virtual array whose elements are located at:

(1) $$r_{\mathrm{virtual}}\left(m,n\right)=\left\{r_{\mathrm{Tm}}+r_{\mathrm{Rn}}|m=1,2,\ldots ,M,n=1,2,\ldots ,N\right\}$$

**Figure 2:**
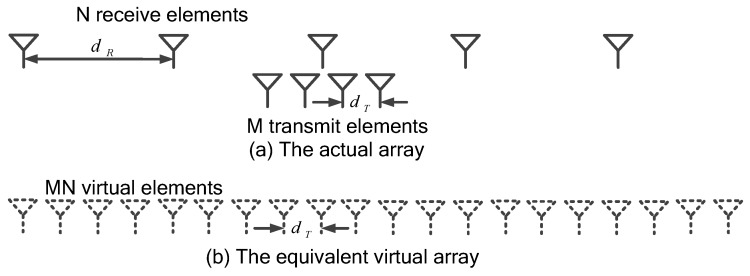
Sketch of the virtual array.

This is the so-called virtual array, which is shown in Figure 2. Correspondingly, the elements in the virtual array are called virtual elements.

### 2.2. Far-Field Condition and Field Division of Array Antennas

In the traditional array theory, array performance is usually analyzed by the antenna pattern which is defined by a target infinitely far away from the antennas. In fact, this kind of ideal target does not exist at all, and a far-field target in engineering should be far enough to guarantee the phase errors of all elements smaller than *p*/8 compared with the ideal one [11]. In conclusion, for an antenna which maximum size is *L*, the far-field distance should be:

(2) $$R_{f}>2L^{2}/\lambda$$

where *R_f_* is the distance from the antenna to the target, *L* is the aperture length of the array, and λ is the wavelength.

**Figure 3:**
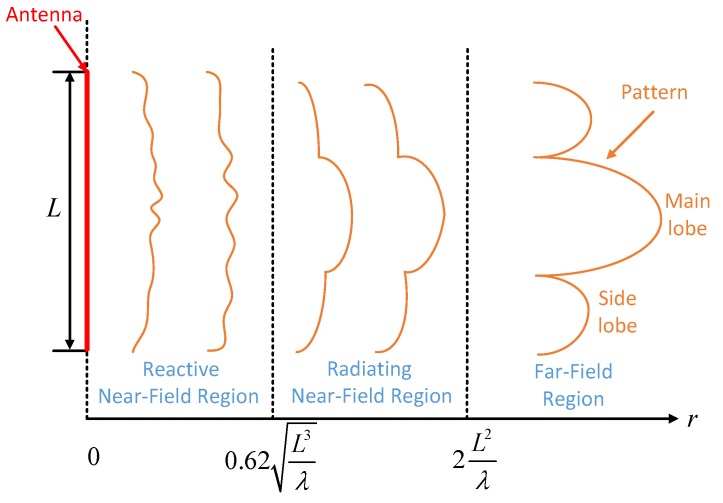
Far-field and near-field region.

Moreover, as shown in Figure 3, the near-field region can still be divided into two parts, the reactive near-field region and the radiating one (Fresnel region). The virtual power of the reciprocating oscillation is greater than the real power transmitted along the radial direction in reactive near-field region [26], thus it is not suitable in the radar application. Therefore, the radiating near-field region is of most importance in this paper, whose distance is limited as follows:

(3) $$0.62\sqrt{\frac{L^{3}}{\lambda }}\leq R\leq 2\frac{L^{2}}{\lambda }$$

## 3. Near-Field PSF of MIMO Imaging Radar

### 3.1. Far-Field Condition and Field Division of MIMO Array

**Figure 4:**
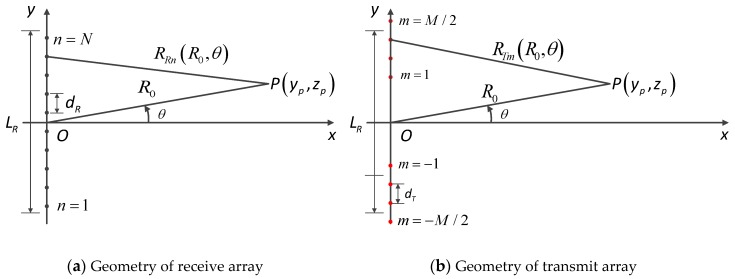
Geometry of MIMO arrays.

Similar to the traditional array radar, the far-field condition of a MIMO radar should be calculated first. As is shown in Figure 4, the target is located with the distance $R_{0}$ and azimuth angle θ from the center of the receive array. Based on the cosine theorem, the target’s distance from the n-th RE, $R_{Rn}\left(R_{0},\theta \right)$, can be expressed as follows:

(4) $$\begin{array}{cc} R_{Rn}\left(R_{0},\theta \right) & =\sqrt{R_{0}^{2}+y_{Rn}^{2}-2y_{Rn}R_{0}\sin\theta }\\ & \approx R_{0}-y_{Rn}\sin\theta +\frac{y_{Rn}^{2}}{2R_{0}}\cos^{2}\theta +\frac{y_{Rn}^{3}}{2R_{0}^{2}}\sin\theta \cos^{2}\theta \end{array}$$

where $y_{Rn}$ is the coordinate values of the n-th RE along y-axis. Similarly, the bistatic distance can be calculated easily as follows:

(5) $$R_{B}\left(R_{0},\theta \right)=2R_{0}-\left(y_{Tm}+y_{Rn}\right)\sin\theta +\frac{y_{Rn}^{2}+y_{Tm}^{2}}{2R_{0}}\cos^{2}\theta +\frac{y_{Tm}^{3}+y_{Rn}^{3}}{2R_{0}^{2}}\sin\theta \cos^{2}\theta$$

where $y_{Tm}$ is the coordinate values of the m-th TE along y-axis, $2R_{0}-\left(y_{Tm}+y_{Rn}\right)\sin\theta$ is equivalent to the bistatic distance of far-field virtual array, and the remaining component is the two-way error, which should be smaller than λ/8 (the two-way error instead of the one-way one):

(6) $$\Delta R_{B}^{far}=\frac{y_{Rn}^{2}+y_{Tm}^{2}}{2R_{0}}\cos^{2}\theta +\frac{y_{Tm}^{3}+y_{Rn}^{3}}{2R_{0}^{2}}\sin\theta \cos^{2}\theta \approx \frac{y_{Rn}^{2}+y_{Tm}^{2}}{2R_{0}}\cos^{2}\theta \leq \frac{\lambda }{8}$$

Considering the largest error at θ=0, the far-field condition of MIMO radar is as follows:

(7) $$R_{far}\geq \frac{4{\left(y_{T}^{\max}\right)}^{2}+4{\left(y_{R}^{\max}\right)}^{2}}{\lambda }$$

In radiating near-field region, the third order term of Equation (5) should be smaller than *l*/8, i.e.:

(8) $$\Delta R_{B}^{Fresnel}=\frac{y_{Tm}^{3}+y_{Rn}^{3}}{2R_{0}^{2}}\sin\theta \cos^{2}\theta \leq \frac{\lambda }{8}$$

The angle with the maximum error, $\theta_{1}=\arctan\left(\sqrt{2}/2\right)$, can be calculated by simple derivation operation. Therefore, the maximum two-way error in Fresnel approximation is $\Delta R_{B}^{Fresnel}\left(\theta =\theta_{1}\right)$, from which the minimum distance of radiating near-field region can be presented as follows:

(9) $$R_{Fresnel}\geq 1.24\sqrt{\frac{{\left(y_{T}^{\max}\right)}^{3}+{\left(y_{R}^{\max}\right)}^{3}}{\lambda }}$$

Combining Equations (7) and (9) yields:

(10) $$1.24\sqrt{\frac{{\left(y_{T}^{\max}\right)}^{3}+{\left(y_{R}^{\max}\right)}^{3}}{\lambda }}\leq R_{Fresnel}<\frac{4{\left(y_{T}^{\max}\right)}^{2}+4{\left(y_{R}^{\max}\right)}^{2}}{\lambda }$$

**Figure 5:**
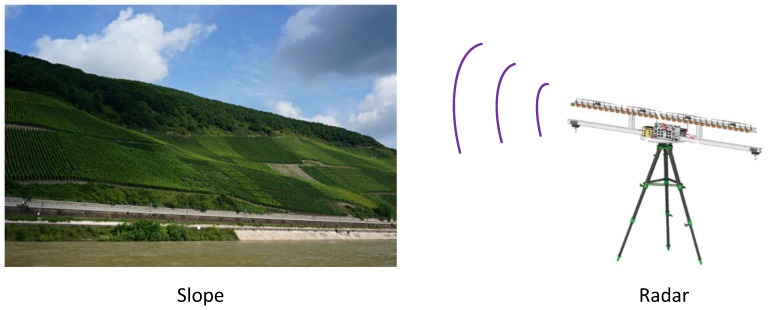
The geometry of slope monitoring.

For the Ku-band ground-based MIMO imaging radar, the array length is in the meter level, so the far-field distance is about several hundred meters and the maximum reactive near-field region is about ten meters. The imaging scene of the slope monitoring radar shown in Figure 5 has a wide depth from tens of meters to thousands of meters, hence there is a need for both far-field and radiating near-field imaging. In this paper, the near field represents the radiating near-field region instead of the reactive one without emphasis.

### 3.2. Definition and Characteristic of Near-Field Pattern

As a generally used tool in evaluating the performance of imaging radar systems, PSF (point spread function) can be treat as the basis of near-field pattern. Considering the PSF can be divided into range dimension and angular dimension, angular PSF can be defined as the profile at the same distance of PSF. With the Parseval’s theorem, the angular PSF can be expressed as:

(11) $$\begin{array}{cc} \chi \left(\theta |R_{0},\theta_{0}\right) & =\sum_{m=1}^{M}\sum_{n=1}^{N}\int_{t}A_{Tm}^{\theta_{0}}\cdot A_{Rn}^{\theta_{0}}\cdot s\left(t-\tau_{mn}^{\theta_{0}}\right)\cdot s^{*}\left(t-\tau_{mn}^{\theta }\right)\;dt\\ & =\int_{f}\left\{P_{s}\left(f\right)\cdot \sum_{m=1}^{M}\left[A_{Tm}^{\theta_{0}}\cdot \exp\left(j2\pi f\left(\tau_{Tm}^{\theta_{0}}-\tau_{Tm}^{\theta }\right)\right)\right]\cdot \sum_{n=1}^{N}\left[A_{Rn}^{\theta_{0}}\cdot \exp\left(j2\pi f\left(\tau_{Rn}^{\theta_{0}}-\tau_{Rn}^{\theta }\right)\right)\right]\right\}\;df \end{array}$$

where $\tau_{mn}^{\theta }$ represents the two-way delay of the target at $\left(R_{0},\theta \right)$ (the distance to the center of receive array is $R_{0}$, and the azimuth angle is θ) when the m-th TE and n-th RE are working. Similarly, $\tau_{Tm}^{\theta }$ and $\tau_{Rn}^{\theta }$ present the one-way delays to the m-th TE and the n-th RE, respectively. Moreover, $A_{Tm}^{\theta_{0}}$ and $A_{Rn}^{\theta_{0}}$ refer to the one-way distance attenuations of the m-th TE and the n-th RE, i.e.:

(12) $$\{\begin{array}{l} A_{Tm}^{\theta_{0}}={\left(4\pi c\tau_{Tm}^{\theta_{0}}\right)}^{-1}={\left(4\pi R_{Tm}^{\theta_{0}}\right)}^{-1}\\ A_{Rn}^{\theta_{0}}={\left(4\pi c\tau_{Rn}^{\theta_{0}}\right)}^{-1}={\left(4\pi R_{Rn}^{\theta_{0}}\right)}^{-1} \end{array}$$

where $R_{Tm}^{\theta_{0}}$ and $R_{Rn}^{\theta_{0}}$ is the one-way distance to the m-th TE and the n-th RE, respectively. It is obvious that almost all the mentioned values in Equation (11) change with $R_{0}$, thus the angular PSF is dependent on the distance of the target. $P_{s}\left(f\right)$ is the power spectral density of the transmitted signal.

It is easily acquired from Equation (11) that the angular PSF can be obtained by integration in the frequency domain with the weight $P_{S}\left(f\right)$. Define the near-field patterns of T/R arrays as:

(13) $$\{\begin{array}{l} F_{T}\left(\theta |R_{0},\theta_{0}\right)=\sum_{m=1}^{M}\left[A_{Tm}^{\theta_{0}}\cdot \exp\left(j2\pi f\left(\tau_{Tm}^{\theta_{0}}-\tau_{Tm}^{\theta }\right)\right)\right]\\ F_{R}\left(\theta |R_{0},\theta_{0}\right)=\sum_{n=1}^{N}\left[A_{Rn}^{\theta_{0}}\cdot \exp\left(j2\pi f\left(\tau_{Rn}^{\theta_{0}}-\tau_{Rn}^{\theta }\right)\right)\right] \end{array}$$

Then the pattern of the MIMO array can be expressed as the product of T/R arrays’ patterns regardless of whether the target is in the near or far field, which is the theoretical basis for the following analysis. Considering the commonly used transmit signal’s power spectral density is square window function, the angular PSF is described as follows:

(14) $$\chi \left(\theta |R_{0},\theta_{0}\right)=\int_{f_{c}-B/2}^{f_{c}+B/2}F_{T}\left(\theta |R_{0},\theta_{0}\right)\cdot F_{R}\left(\theta |R_{0},\theta_{0}\right)\;df$$

## 4. Non-Collinear MIMO Array Design for Near-Field Imaging

As is mentioned in the introduction, it is unavoidable to consider the defocus caused by the height difference between the T/R arrays and the grating lobes caused by the non-uniform virtual array. First, for good focus of the whole scene, a design method of the height difference is proposed. Second, a method to adjust the far-field MIMO arrays is presented, by which the grating lobes are effectively suppressed.

### 4.1. Design of the Height Difference between T/R Arrays

The virtual array theory assumes that all the array elements are located along the same line. However, due to the physical size of the antennas and the coupling of the T/R elements, a certain height difference between the T/R arrays is inevitable.

**Figure 6:**
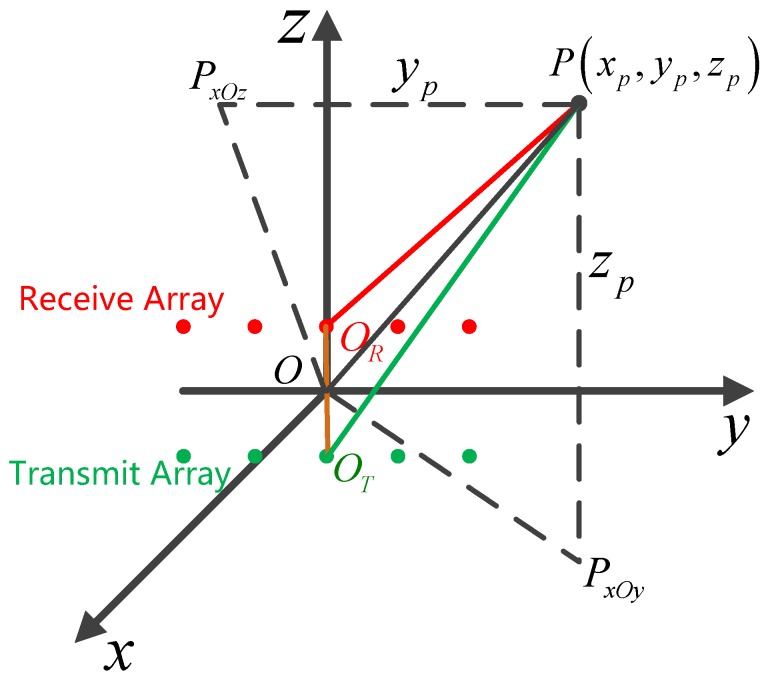
3D Geometric model of MIMO imaging radar array.

As is shown in Figure 6, it is assumed that the transmit array is lower than the receive array and the height difference marked by a brown line is Δh. Establish a coordinate system with the average of the T/R arrays’ centers as the origin O. In this coordinate system, T/R arrays are in the y-O-z plane and both parallel to y-axis, thus TEs are located at $\left(0,y_{Tm},-\Delta h/2\right)$ and REs are located at $\left(0,y_{Rn},\Delta h/2\right)$. Considering a target at $P\left(x_{p},y_{p},z_{p}\right)$, it is located at $\left(r_{p},\theta_{p},\phi_{p}\right)$ in the spherical coordinate system ($r_{p}=\left|\vec{OP}\right|,\theta_{p}=\langle \hat{x},\vec{OP_{xOy}}\rangle ,\phi_{p}=\langle \vec{OP_{xOy}},\vec{OP}\rangle$) and its azimuth angle is $\theta_{cen}=\langle \vec{OP_{xOz}},\vec{OP}\rangle$, where 〈⋅〉 is the angle operator of two vectors, $P_{xOz}$ and $P_{xOy}$ are the projection points of P in x-O-z and x-Oy plane, and $\hat{x}$ is the unit vector in x-axis direction. Mark the centers of T/R arrays as $O_{T}$ and $O_{R}$, respectively, then the distance from the target to the center of receive array is $r_{R,p}=\left|\vec{O_{R}P}\right|$ and the azimuth angle to the receive array is $\theta_{R,cen}=\langle \vec{O_{R}P_{xOz}},\vec{O_{R}P}\rangle$. Similarly, the distance of transmit array is $r_{T,p}=\left|\vec{O_{T}P}\right|$ and the azimuth angle to the transmit array is $\theta_{T,cen}=\langle \vec{O_{T}P_{xOz}},\vec{O_{T}P}\rangle$. When the target is with the average height of the T/R arrays, it is obvious that:

(15) $$r_{T,p}=r_{R,p},\;\theta_{T,cen}=\theta_{R,cen}$$

**Figure 7:**
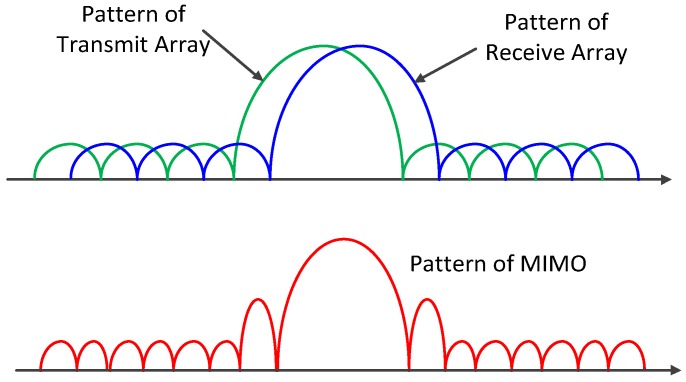
Influence of peaks’ offset of T/R patterns.

However, Equation (15) is no longer valid when the target’s height does not equal to the average height of T/R arrays. In this case, the peak position of the T/R patterns will shift to a certain extent from the ideal position $\sin\theta_{T,cen}=\sin\theta_{R,cen}$ to $\sin\theta_{T,cen}\neq \sin\theta_{R,cen}$, resulting in high side lobes in the azimuth angle dimension. As is shown in Figure 7, the offset leads to the increase of PSLR, so the PSLR will be selected as the imaging reference in the following analysis.

#### 4.1.1. Peaks’ Offset of T/R Patterns

The geometric relationship in $\Delta O_{T}P_{xOz}P$ shows:

(16) $$y_{p}=r_{T,p}\cdot \sin\theta_{T,cen}$$

and:

(17) $$y_{p}=r_{R,p}\cdot \sin\theta_{R,cen}=r_{p}\sin\theta_{cen}$$

Combining Equations (16) and (17) yields:

(18) $$\{\begin{array}{l} \sin\theta_{T,cen}=\frac{r_{p}}{r_{T,p}}\sin\theta_{cen}\\ \sin\theta_{R,cen}=\frac{r_{p}}{r_{R,p}}\sin\theta_{cen} \end{array}$$

Therefore, the peaks’ offset is:

(19) $$\left|\delta \sin\theta_{cen}\right|=\left|\sin\theta_{R,cen}-\sin\theta_{T,cen}\right|=\left|\frac{1}{r_{R,p}}-\frac{1}{r_{T,p}}\right|r_{p}\left|\sin\theta_{cen}\right|$$

which implies that the offset is proportional to $\left|\sin\theta_{cen}\right|$. Thus, the defocusing deteriorates as the azimuth angle of the target increases.

#### 4.1.2. Difference of Distances from the Target to TEs/REs

Applying the cosine theorem in $\Delta OO_{T}P$ and $\Delta OO_{R}P$ yields:

(20) $$r_{T,p}=\sqrt{{\left(r_{p}+\frac{\Delta h}{2}\sin\left(\phi_{p}\right)\right)}^{2}+\frac{\Delta h^{2}}{4}\cos^{2}\left(\phi_{p}\right)}$$

and:

(21) $$r_{R,p}=\sqrt{{\left(r_{p}-\frac{\Delta h}{2}\sin\left(\phi_{p}\right)\right)}^{2}+\frac{\Delta h^{2}}{4}\cos^{2}\left(\phi_{p}\right)}$$

Combining Equations (19)–(21), the peaks’ offset can be expressed as $\delta \sin\theta_{cen}\left(r_{p},\theta_{cen},\phi_{p};\Delta h\right)$, and:

(22) $${\delta \sin\theta_{cen}|}_{\theta_{p}=0}={\delta \sin\theta_{cen}|}_{\theta_{cen}=0}=0,\;\forall r_{p}$$

Equation (22) means that targets with the average height of T/R arrays won’t defocus, no matter if in the near-field area or the far-field area. Meanwhile, it is easy to prove that:

(23) $$\frac{\partial }{\partial r_{p}}\left|\delta \sin\theta_{cen}\right|<0,\;\left|r_{p}\sin\phi_{p}\right|>\frac{\Delta h}{2}$$

and:

(24) $$\frac{\partial }{\partial \left|\phi_{p}\right|}\left|\delta \sin\theta_{cen}\right|>0,\;\phi_{p}\neq 0$$

Therefore, the offset raises with the decrease of distance and the increase of elevation angle. Thus, it is only necessary to determine the offset of the edge point target at the closest distance, the maximum azimuth angle and the maximum elevation angle. If the imaging performance of the edge point meets the design requirement, the height difference is acceptable.

Accordingly, there is:

(25) $$\frac{\partial }{\partial \Delta h}\left|\delta \sin\theta_{cen}\right|>0$$

The offset increases as the array’s height difference increases. As a conclusion, the maximum peaks’ offset within the observed scene is:

(26) $${\left|\delta \sin\theta_{cen}\right|}_{\max}=\left|\delta \sin\theta_{cen}\right|\left(r_{p\_\min},\theta_{cen\_\max},\phi_{p\_\max};\Delta h\right)$$

It is ensured that all targets within the imaging scene won’t defocus as long as the offset is smaller than the maximum non-defocus offset. It should be noted that the maximum offset does not always mean the worst imaging performance because the PSLR is not a monotonically variable of the pattern when the offset is larger than the resolution.

#### 4.1.3. Solution of Maximum Height Difference

We denote the azimuth resolutions of T/R arrays at edge points as $\sigma_{T}\left(\sin\theta_{cen}\right)$ and $\sigma_{R}\left(\sin\theta_{cen}\right)$ respectively. To ensure the main lobe does not split, the maximum offset should not exceed the resolution, i.e.:

(27) $${\left(\delta \sin\theta_{cen}\right)}_{\max}\leq \min\left\{\sigma_{T}\left(\sin\theta_{cen}\right),\sigma_{R}\left(\sin\theta_{cen}\right)\right\}$$

Meanwhile, we can get the relation curve of edge point’s azimuth PSLR with maximum offset, by which the maximum acceptable offset ${\left(\delta \sin\theta_{cen}\right)}_{\max}$ can be calculated. Then the maximum acceptable height difference is:

(28) $$\Delta h_{\max}=\underset{\Delta h}{\arg}\left\{\delta \sin\theta_{cen}\left(r_{p\_\min},\theta_{cen\_\max},\phi_{p\_\max};\Delta h\right)={\left(\delta \sin\theta_{cen}\right)}_{\max}\right\}$$

### 4.2. Design of Low Grating Array for Near-Field MIMO Imaging

**Figure 8:**
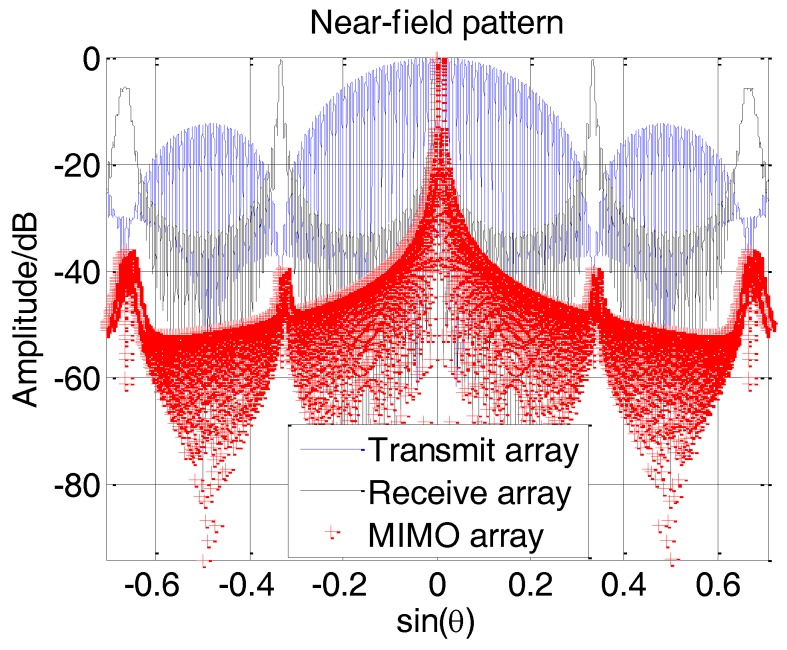
Grating lobes in near-field pattern of MIMO array.

Under the far-field condition, the grating lobes of the sparse array’s pattern are canceled out by the nulls of the dense array’s pattern completely and no grating lobes appear. However, both the dense and the sparse arrays’ patterns will produce significant distortions under near-field conditions, resulting in grating lobes on the MIMO array’s pattern. Figure 8 shows the near-field pattern at 30 m of a MIMO array with an equivalent virtual array length of about 4.75 m, in which the grating lobes are obvious.

**Figure 9:**
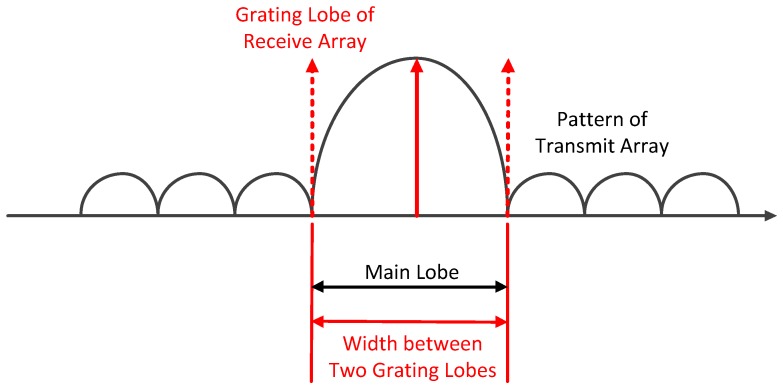
Description of near-field low grating lobe array.

Based on the patterns’ multiplication principle above, we add a window to the dense array to suppress side lobes and increase the number of the dense elements to reduce the width of the pattern’s main lobe, which can ensure grating lobes of the sparse arrays pattern are all located in the side lobe regions of the dense arrays pattern. The principle of this method is shown in Figure 9. Since the main influence of the non-collinear array on the angular PSF is the slight offset of the peaks, to which the proposed method is insensitive, we can treat the non-collinear array as a collinear one directly in suppressing the grating lobes.

After designing a MIMO array for far-field targets, a near-field low grating lobe MIMO array can be obtained by two steps. If the required peak-to-peak grating lobe ratio is $\sigma_{GL}$ in dB, the specific steps are as follows:

#### 4.2.1. Suppression Caused by the Bandwidth

It is easy to know that the positions of grating lobes are related to the frequency, so the existence of bandwidth can smooth the grating lobes in the near-field area. Because this effect $\sigma_{B}$ (gain in dB provided by bandwidth) is difficult to obtain by theoretical analysis, we calculate it by simulation of edge points’ imaging performance.

#### 4.2.2. Adjust the Dense Array

After considering the effect of bandwidth, the dense array will be adjusted to suppress the grating lobes. Firstly, a window function w(n) with the side lobe level of $\sigma_{GL}+\sigma_{B}$ should be added to the dense array to get a low-side-lobes pattern, where $\sigma_{GL}$ is the maximum acceptable level of grating lobes in dB. With this window function, the main lobe of the dense array will be widened so that the grating lobes of the sparse array cannot be completely covered by the side lobes of the dense array. Therefore, it is necessary to extend the length of the dense array. If the expansion factor of the first null after adding window function is ξ, the extended number of TEs should be:

(29) $$N_{T}^{\prime }=\mathrm{round}\left(N_{T}/\xi \right)$$

## 5. Simulations and Experiment

### 5.1. Simulations

**Figure 10:**
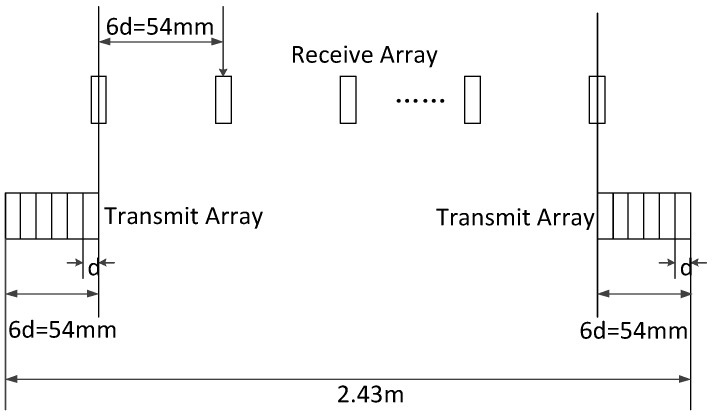
Structure of MIMO array in simulations.

To verify the validity of the proposed design methods, this section includes two simulations performed using Matlab. The adopted far-field MIMO array is shown in Figure 10. The transmit array is a dense ULA, and is divided into two sub-arrays placed at both ends of the sparse receive ULA. The parameters used in the simulations are shown in Table 1.

#### 5.1.1. Simulation of Non-Collinear Array’s Height Difference Design

In the used MIMO array shown in Figure 10 there is a certain height difference Δh between T/R arrays. To achieve the requirements of imaging, the edge point should be chosen at (30 m,45°,22.5°) in the coordinate shown in Figure 2. It is easy to calculate the resolution:

(30) $$\sigma_{T}\left(\sin\theta_{cen}\right)\approx \sigma_{R}\left(\sin\theta_{cen}\right)=0.886\frac{\lambda }{N_{R}d_{R}}=0.0067$$

Therefore, the maximum offset should be:

(31) $${\left(\delta \sin\theta_{cen}\right)}_{\max}\leq 0.0067$$

**Figure 11:**
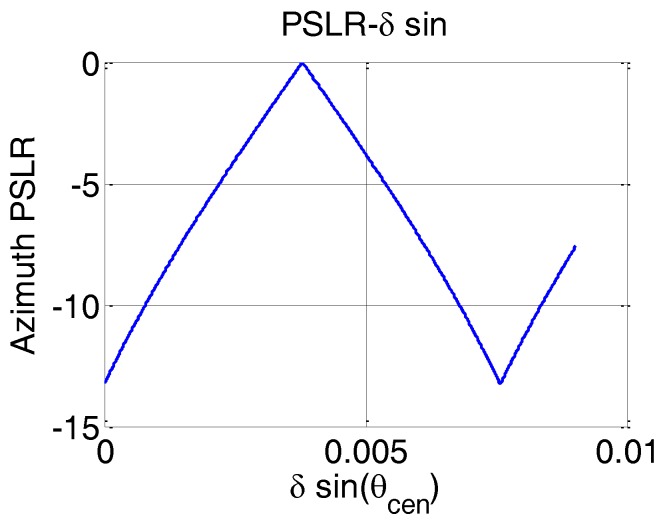
Relationship between PSLR and offset.

It has derived in [26] that the distance attenuation can be treated as a constant ${\left(4\pi R_{0}\right)}^{-1}$ in far-field and radiating near-field region, thus can be ignored in the following analysis. Furthermore, close to the main lobe, the near-field pattern equals to the far-field pattern approximately, i.e.:

(32) $$\left|F_{T}\left(\theta \right)\right|=\left|\frac{\sin\left(\frac{\pi }{\lambda }\frac{N_{T}}{2}d_{T}\left(\sin\theta -\sin\theta_{T,cen}\right)\right)}{\sin\left(\frac{\pi }{\lambda }d_{T}\left(\sin\theta -\sin\theta_{T,cen}\right)\right)}\cdot \cos\left(\frac{2\pi }{\lambda }y_{Tcen}\left(\sin\theta -\sin\theta_{T,cen}\right)\right)\right|$$

and:

(33) $$\left|F_{R}\left(\theta \right)\right|=\left|\frac{\sin\left(\frac{\pi }{\lambda }N_{R}d_{R}\left(\sin\theta -\sin\theta_{R,cen}\right)\right)}{\sin\left(\frac{\pi }{\lambda }d_{R}\left(\sin\theta -\sin\theta_{R,cen}\right)\right)}\right|$$

where $y_{Tcen}$ is the distance from the center of receive array to the center of transmit sub-array. The relationship between PSLR and the offset is shown in Figure 11.

**Figure 12:**
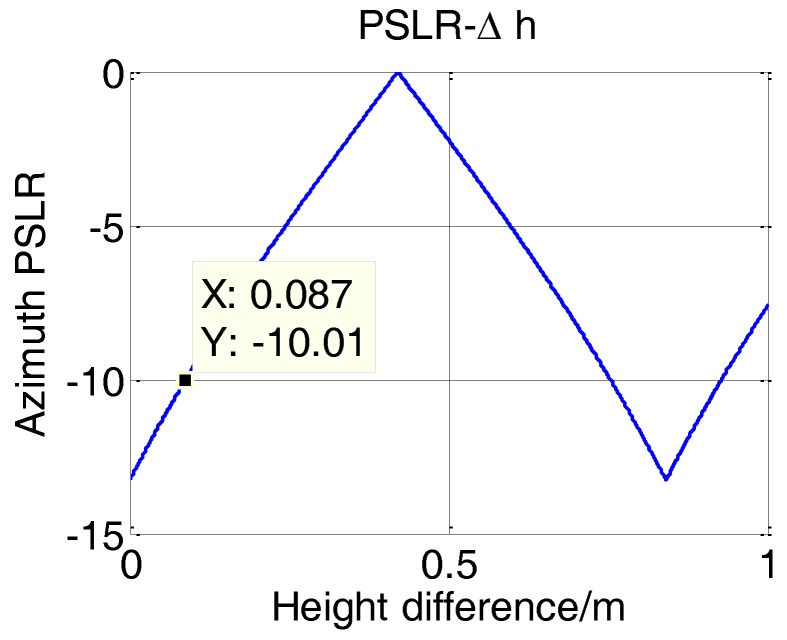
Relationship between PSLR and height difference.

Meanwhile, by substituting the coordinates of the edge point into Equations (19)–(21), the relationship between the offset and the height difference can be obtained. Combining these two curves, we can get the curve between PLSR and the height difference, which is shown in Figure 12.

**Figure 13:**
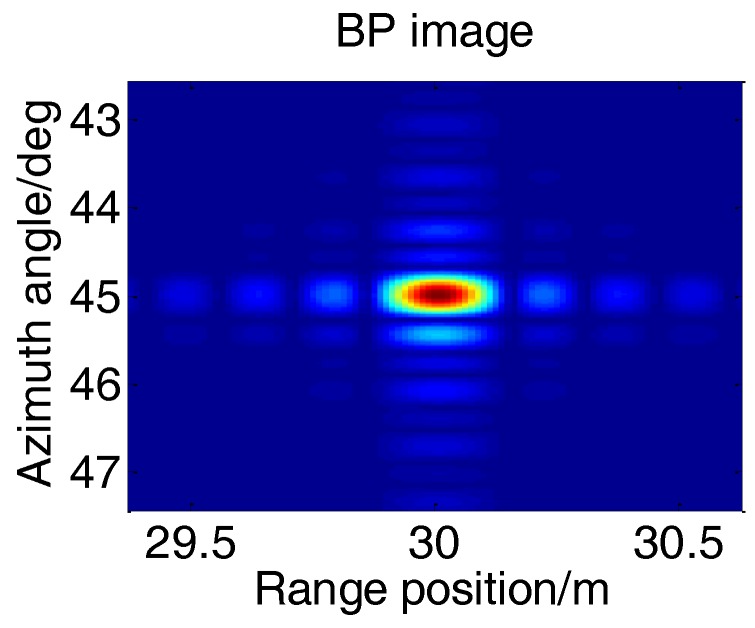
Image of edge point.

Taking into account the effective isolation between the T/R elements, the height difference between T/R arrays should be guaranteed in the sub-meter scale. Select −10 dB as the maximum acceptable PSLR without any window function, then the maximum acceptable height difference can be obtained as 8.7 cm, with which the imaging result of edge point can be shown in Figure 13.

**Figure 14:**
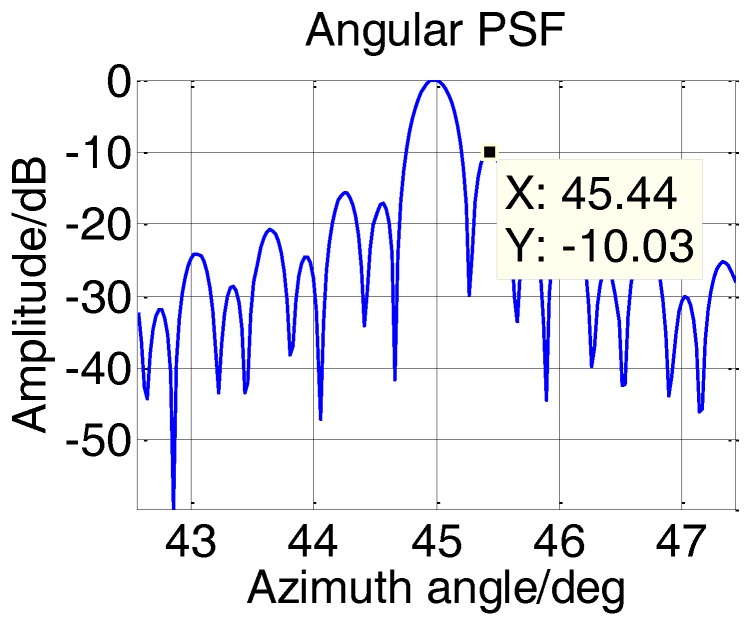
Angular PSF of edge point.

Figure 14 shows the angular PSF of edge point, which proved the validity of the height difference design method.

#### 5.1.2. Simulation of Low-Grating-Lobe Near-Field Array Design

It is easy to verify that the MIMO array above has an ideal imaging performance for far-field targets. Nevertheless, high grating lobes will appear in the images of near-field targets, and the array should be modified. The specification of grating lobes is selected as $\sigma_{GL}=-50\;\mathrm{dB}$. Apparently, the edge point should be 30 m from the origin with 45° azimuth angle.

It can be found that $\sigma_{B}\approx 10\;\mathrm{dB}$ by Matlab simulation when the bandwidth is 1 GHz. Thus $\sigma_{GL}+\sigma_{B}=-40\;\mathrm{dB}$ is used to design the window function. If a Taylor window is chosen, the expansion factor should be ξ=1.819. Taking into consideration the existence of two transmit sub-arrays, $N_{T}^{\prime }$ should be an even number, i.e.:

(34) $$N_{T}^{\prime }=2\cdot \mathrm{round}\left(N_{T}/2\cdot \xi \right)=22$$

**Figure 15:**
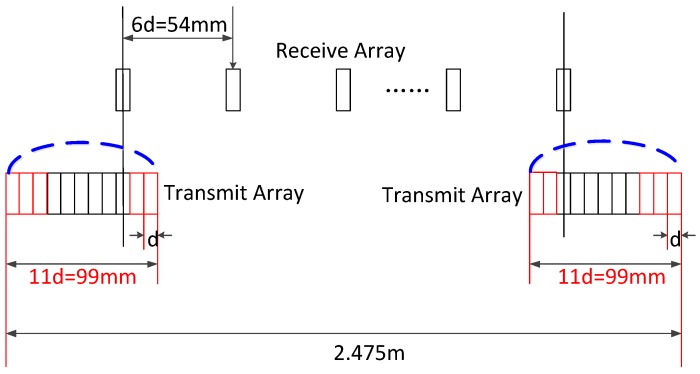
MIMO array with low grating lobes in near-field area.

**Figure 16:**
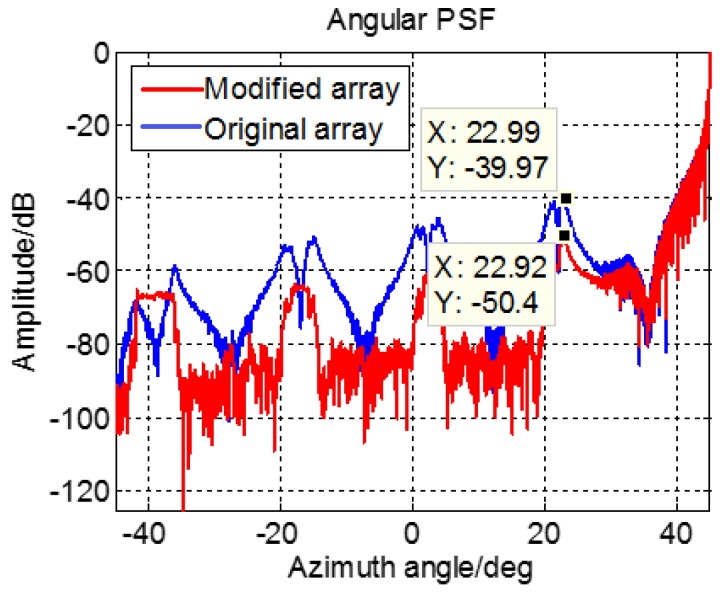
Angular PSF of modified MIMO array.

Adjusting the transmit array without changing the interval between two nearby elements, we can get the new array shown in Figure 15. The angular PSF of the edge point is shown in Figure 16, from which the level of maximum grating lobe is −50.4 dB. Obviously, the validity of the proposed method is verified by this simulation.

### 5.2. Experiment

**Figure 17:**
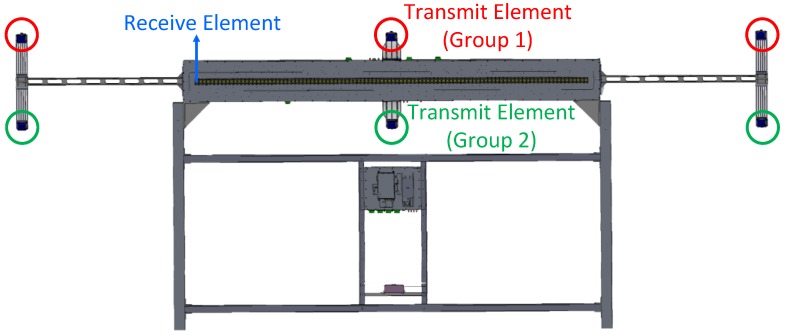
MIMO radar system for the experiment.

The MIMO array used in the experiment with the parameters listed in Table 2 is shown in Figure 17, in which the TEs are labeled by red circles and green circles while one of the 96 REs is labeled by a blue arrow. Although there are six TEs in this system, only the three TEs in group 1 labeled by red circles are used. If all TEs are processed together, the height of targets can be measured by interference technology, which is beyond the scope of this paper.

**Figure 18:**
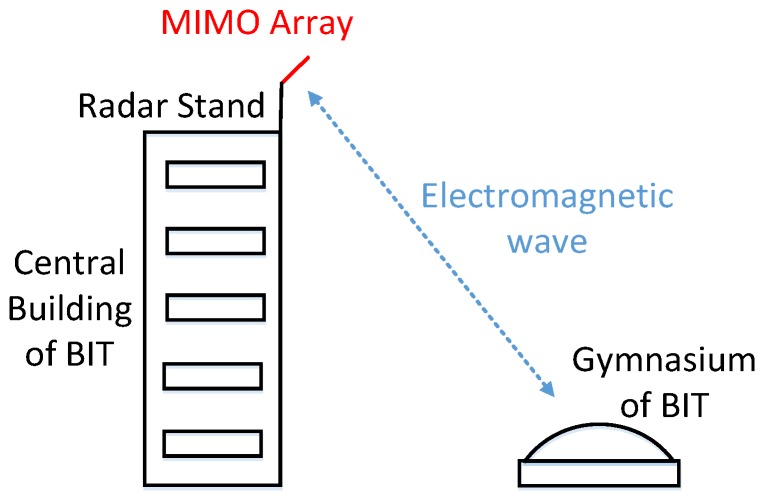
Geometry of this experiment.

**Figure 19:**
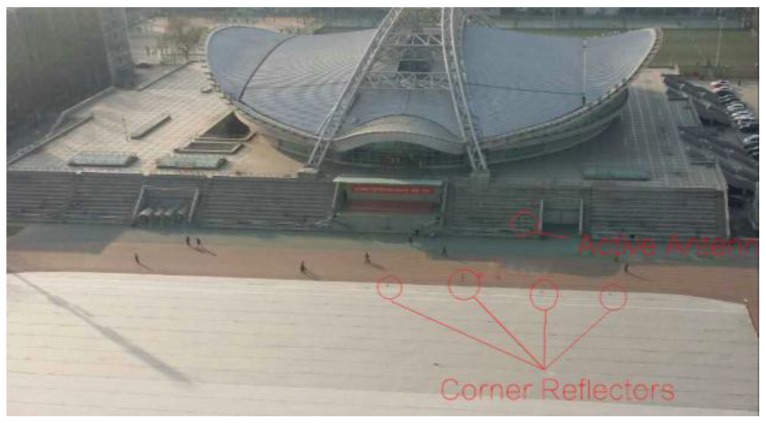
Imaging scene of this experiment.

This experiment was performed in Beijing Institute of Technology (BIT). As is shown in Figure 18, the radar was arranged at the top of the central building, and the antenna was directed at the gymnasium. It should be noted that the arrangement of the array antenna is perpendicular to the observation surface in Figure 18, and the red line is the baseline of the two groups of TEs. Figure 19 shows the scene of this experiment. The main building in the scene is the gymnasium of BIT, in front of which there are four corner reflectors and an active antenna.

**Figure 20:**
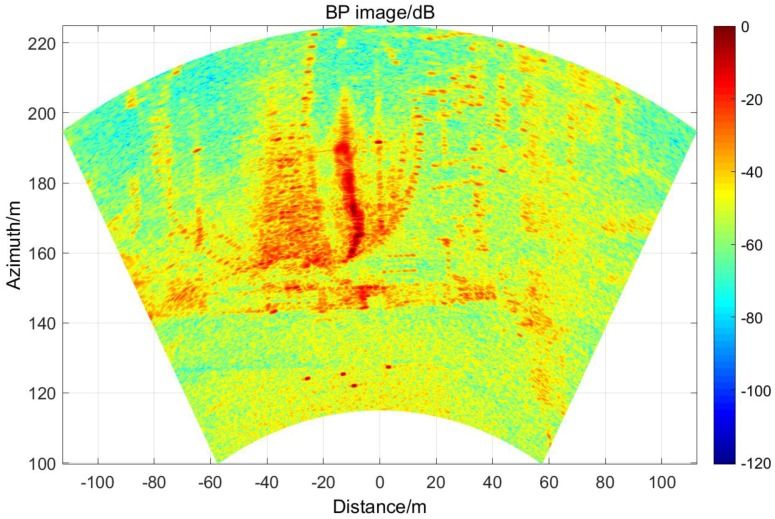
Image of the scene.

With three TEs and 96 REs, there are 288 individual observation channels in this system. After processing the echoes by BP (backward projection) algorithm, a radar image in dB can be obtained, shown in Figure 20. From this picture, the outline of the gymnasium, the platform below the gymnasium and four point targets corresponding to the corner reflectors can be clearly seen. The length of the gymnasium is about 100 m and the distances from the array to the corner reflectors are about 120~130 m. In particular, the active antenna does not appear in the image because it is placed for calibration and closed after that.

**Figure 21:**
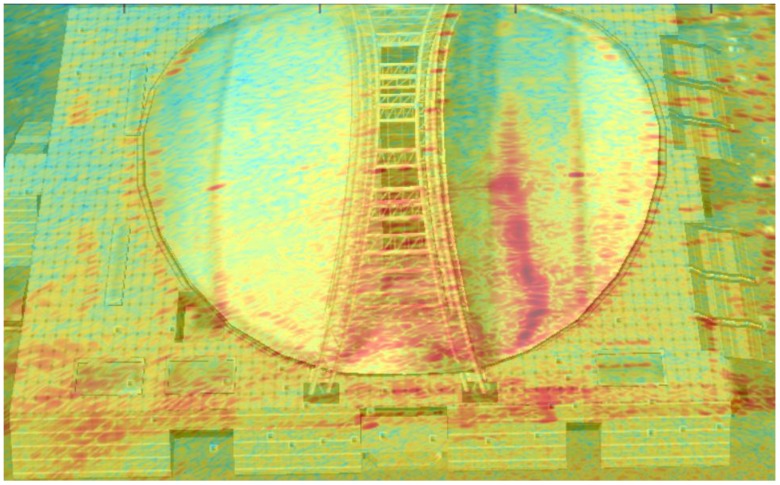
Comparison of optical picture and radar image.

To prove the correctness of the imaging results, we compared the optical images obtained from Google Earth and the radar image in this experiment in Figure 21. It obvious that the outline of the gymnasium in these two pictures can match to each other perfectly.

**Figure 22:**
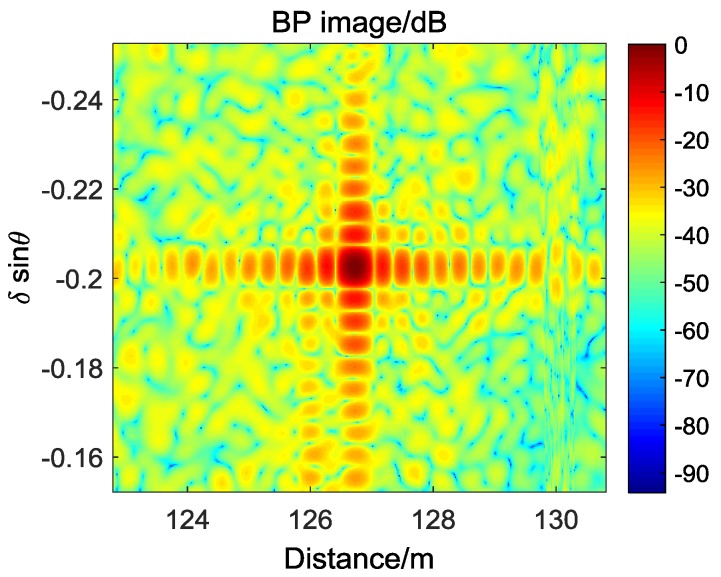
Imaging of corner reflector.

**Figure 23:**
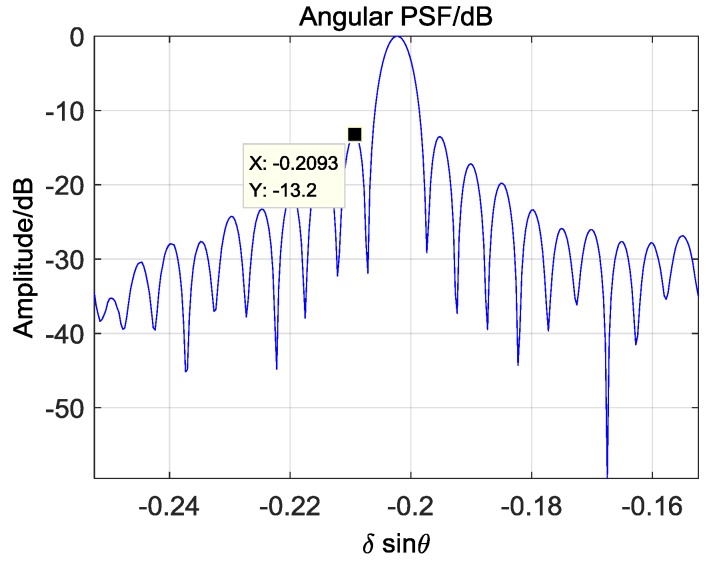
Angular PSF of corner reflector.

Although the overall imaging performance has been verified by outline comparison, the specific parameters of the point target imaging still need to be analyzed. In former BP images, two-dimensional Hamming window are added for better focus, but it is not necessary in the following analysis of point target imaging. The BP image of the left corner reflector without window function is shown in Figure 22 and its angular profile at the maximum value is presented in Figure 23.

**Figure 24:**
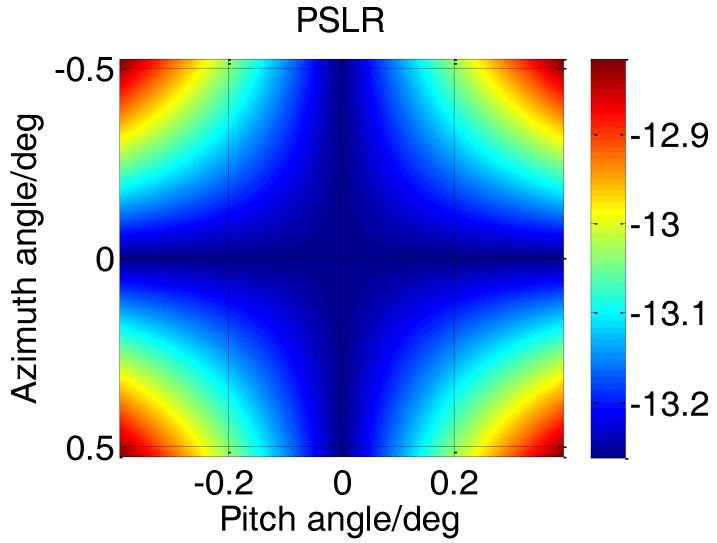
PSLR at shortest distance.

Figure 24 shows the relationship between PSLR and the two-dimensional angles at 115 m. It means that the performance of all targets in the scene should be better than −12.8 dB. Specially, the PSLR in Figure 23 is −13.2 dB and matches the theoretical performance well.

## 6. Conclusions

This paper has discussed defocus and high grating lobe problems in the azimuth dimension of non-collinear MIMO imaging radar systems in the near-field area. Firstly, the near-field pattern is defined by the concept of PSF, with which the mentioned two problems are explained. Secondly, a method based on numerical simulation of PSLR is proposed to design the height difference of the non-collinear MIMO array. Thirdly, a MIMO array design method is presented, which can realize low grating lobes in the near-field area by adjusting the TEs’ position and adding TEs. Finally, two Matlab simulations and an experiment are performed to verify the validity of the two proposed methods. The simulations show that the proposed methods can design non-collinear MIMO arrays accurately, while the experiment proves that the designed MIMO array with a small height difference can provide a good imaging result for a monitored scene.

## Figures and Tables

**Table 1:** Parameters of simulations.

Parameters	Values	Parameters	Values
Wavelength λ	0.018 m	Bandwidth B	1 GHz
Duration T_p_	5 μs	Sample rate fs	1.2 GSa/s
Number of TEs N_T_	12	Interval between TEs d_T_	9 mm
Number of REs N_R_	44	Interval between REs d_R_	54 mm
Distance	30~3000 m	Azimuth angle	−π/4~π/4 rad
Elevation angle	−π/8~π/8 rad		

**Table 2:** Parameters of the Experimental MIMO Radar System.

Parameters	Values	Parameters	Values
Wavelength λ	3.16 cm	Bandwidth B	480 MHz
Number of TEs N_T_	3	Interval between TEs d_T_	2.112 m
Number of REs N_R_	96	Interval between REs d_R_	2.2 cm
Distance	115~225 m	Azimuth angle	−π/6~π/6 rad
Elevation angle	−π/8~π/8 rad	Height difference	0.25 m
